# Predictors of aging out of heavy episodic drinking in emerging adults: a systematic review protocol

**DOI:** 10.1186/s13643-019-1139-9

**Published:** 2019-09-04

**Authors:** Tashia D. Petker, Jillian Halladay, Lana Vedelago, Mark A. Ferro, Jalie Tucker, Mark S. Goldman, James G. Murphy, James MacKillop

**Affiliations:** 1 Department of Psychology, Neuroscience and Behaviour, McMaster University, Hamilton, Ontario Canada; 2 Peter Boris Centre for Addictions Research, St. Joseph’s Healthcare Hamilton/McMaster University, 100 West 5th Street, Hamilton, Ontario Canada; 3 Department of Psychiatry and Behavioural Neurosciences, McMaster University, Hamilton, Ontario, Canada; 4 Department of Health Research Methods, Evidence, and Impact, McMaster University, Hamilton, Ontario Canada; 5 School of Public Health and Health Systems, University of Waterloo, Waterloo, Ontario Canada; 6 Department of Health Education and Behaviour, College of Health and Human Performance, University of Florida, Gainesville, FL USA; 7 Department of Psychology, College of Arts and Sciences, University of South Florida, Tampa, FL USA; 8 Department of Psychology, University of Memphis, Memphis, TN USA

**Keywords:** Alcohol, Binge drinking, Heavy episodic drinking, Alcohol misuse, Emerging adults, Young adults, Alcohol use disorder, Maturing out

## Abstract

**Background:**

Heavy episodic drinking (HED) refers to alcohol consumption that exceeds the recommended threshold for a given episode and increases risk for diverse negative alcohol-related consequences. A pattern of weekly HED is most prevalent in emerging adults (i.e., age 18–25). However, rates of HED consistently decline in the mid to late twenties, referred to as ‘aging out’ or ‘maturing out’ of HED. Although many individual studies have followed changes in drinking behaviour over the transition to adulthood, there has yet to be a systematic review to identify consistent factors contributing to risk (i.e. failure to age out) and protection (i.e. successful aging out). The objective of this review will be to summarize and critically appraise the literature on factors contributing to aging out of HED among emerging adults.

**Methods:**

A systematic search of observational cohort studies following drinking behaviours in age cohorts overlapping with the emerging adulthood period will be conducted in MEDLINE, EMBASE, PsychInfo, and CINAHL. Two independent reviewers will evaluate identified studies for inclusion eligibility, extract study data, and assess the quality of included studies. Primary outcomes will be quantity/frequency of alcohol use (e.g. drinks/week) and severity of alcohol-related problems. Predictors of maturing out of HED will be reported narratively, and where appropriate, random effects meta-analyses will be conducted to provide pooled effect sizes. An evidence map will be created to characterize the overall pattern of findings.

**Discussion:**

This systematic review will provide a timely and warranted summary of published work contributing to understanding aging out of heavy episodic drinking. Our findings will provide critical commentary on the developmental course of HED during the transition from adolescence to adulthood and will be the first review to consider both protective and risk factors for maturing out of frequent binge drinking. By highlighting factors identifying those at-risk for prolonged heavy episodic drinking, our conclusions will have important treatment implications for primary, secondary, and tertiary intervention strategies.

**Systematic review registration:**

PROSPERO CRD42017078436.

**Electronic supplementary material:**

The online version of this article (10.1186/s13643-019-1139-9) contains supplementary material, which is available to authorized users.

## Background

The term ‘heavy episodic drinking’ is used to describe a pattern of alcohol consumption that typically exceeds the recommended threshold for a single drinking episode. Terms that are often used synonymously with heavy episodic drinking (HED) include binge drinking, heavy drinking, risky drinking, and alcohol misuse. A commonly used definition of HED is when 4+ drinks for women and 5+ drinks for men are consumed within a few hours [1], generally reflecting alcohol consumption that brings blood alcohol concentration (BAC) to 0.08 g/dL. The prevalence of weekly HED among drinkers age 15 and older has been estimated at 16% worldwide, 13.7% in North America, and 16.5% in Europe [2]. But, the prevalence of HED is unevenly distributed across age cohorts, as older adults tend to have lower consumption rates than young adults. For example, HED is observed to peak among emerging adults (i.e. age 18–25), with weekly prevalence rates estimated at 41% in Canada [3] and 38.8% in the USA [4].

Importantly, the prevalence of heavy episodic drinking from adolescence into adulthood is curvilinear, with alcohol initiation typically in adolescence, sharp increases in heavy drinking from age 18–22, and then consistent decreases over the course of the twenties [5]. This trajectory indicates that during emerging adulthood many people naturally ‘age out’ or ‘mature out’ of excessive drinking, although others do not and continue to drink at risky levels or exhibit a further progression [6]. Determining risk factors for longstanding heavy episodic drinking is therefore critical for improving identification of at-risk individuals and facilitating early intervention.

Several risk factors for persistent heavy episodic drinking have been extensively examined in individual studies [6–8], but the mechanisms for maturing out are still far from fully understood [9, 10]. The most well-understood predictors of changes in problematic drinking in emerging adults include role transitions and life events. Beginning full-time employment, getting married, and having children are associated with decreases in drinking, while other milestones such as starting post-secondary education and divorce have been found to predict increases in drinking [11, 12]. More recent evidence implicates factors other than role transitions in the maturing out process, such as personality traits, drinking motives, and alcohol expectancies. For instance, a recent review identified consistent associations between traits related to impulsivity (i.e. sensation-seeking, positive and negative urgency, reward sensitivity, lack of perseverance/premeditation) and heavy drinking in emerging adults [13]. Recent longitudinal findings suggest that the influence of role transition and personality on drinking behaviour changes over the course of young adulthood, such that in early young adulthood role transitions are more influential but later personality plays a greater role [14, 15]. In addition, the expectancies an individual has for the predicted effects of alcohol may also influence how much and how often they drink. Positive expectancies of consuming alcohol in adolescence have been found to predict greater alcohol consumption and HED in adulthood [8]; however, a need for more prospective studies has been identified to better understand this trajectory over emerging adulthood [16].

The empirical support for the preceding predictors largely comes from individual cross-sectional and longitudinal studies, which further highlights the need for collective analysis of studies predicting changes in drinking during the transition into full adulthood. In addition to impulsivity, drinking motives, and other factors mentioned above, we also intend to explore the literature for other factors that may contribute to the maturing out process. Distal factors of interest include family history of alcohol use and related problems, genetic markers, childhood experiences, and temperament. Commonly measured proximal factors will also be evaluated, including peer influence, antisocial traits and behaviour, religiosity, and current psychiatric issues. We will also examine newly emerging areas of inquiry that have not yet been systematically reviewed in the context of maturing out of heavy episodic drinking. These factors include implicit cognitive biases, executive function, and behavioural economic indices (e.g. delay discounting, reinforcing value of alcohol). Taken together, we hope to provide a critical commentary on the state of the literature predicting maturing out of HED in emerging adulthood and to identify areas in need of further study.

The primary objective of this systematic review is to summarize the existing literature on predictors of maturing out of heavy episodic drinking in emerging adulthood by identifying and evaluating the current evidence (participants = emerging adults; interventions = passage of time; comparator = predictor variables; outcomes = drinking).

Specifically, our aims are as follows:

1. Characterize the primary source peer-reviewed empirical studies using longitudinal designs to measure changes in drinking trajectories across emerging adulthood. Within this body of literature, we aim to categorize and describe factors predicting changes in heavy episodic drinking and to further parse these factors based on their role of risk (i.e. prediction, persistence, or exacerbation) and protection (i.e. prediction of attenuation or cessation).
2. Present an evidence map of the findings according to predictors to synthesize the literature in a user-friendly format.
3. Evaluate the quality of existing studies.
4. Where sufficient studies are present, quantitatively synthesize the associations between risk/protective factors and drinking outcomes.
5. Provide a critical appraisal of existing literature and identify substantive and methodological gaps requiring further research.

## Methods

The present protocol has been registered within the PROSPERO database (CRD42017078436) and will being reported in accordance with the reporting guidance in the Preferred Reporting Items for Systematic Reviews and Meta-Analyses Protocols (PRISMA-P) statement [17] (see checklist in Additional file 1).

### Search strategy

Existing primary research will be identified and retrieved from OVID MEDLINE In-Process (1946 to present), EMBASE (1974 to present), PsycINFO (1806 to present), and CINAHL databases. The search will involve all related subject headings and key terms associated with emerging adulthood (e.g. young adult* OR emerging adult* OR student OR adolecsen*), alcohol use, and longitudinal designs (e.g. longitudinal, growth mixture modeling, group-based trajectory modeling). An example is provided in Additional file 2. The final search strategy will be developed in collaboration with a university librarian to ensure a maximally inclusive search strategy; the search will be initially mapped in EMBASE and then adjusted for other databases. We will also manually explore the citations of included primary studies and reviews to identify relevant articles omitted by search results, and contact authors for further information and data when appropriate. Only investigations published in English will be included.

### Inclusion/exclusion criteria

We will include published peer-reviewed longitudinal studies that examine drinking behaviours in emerging adults (age 18–25), including study samples that overlap with emerging adulthood (e.g. adolescents followed age 14–19; emerging adults followed age 22–27). Studies included will examine human subjects and will minimally include measures of alcohol use (severity and/or consumption). Studies that also measure non-alcohol psychoactive drug use will be included if they report drinking behaviour. To focus the scope of the review to predictors of aging out of heavy episodic drinking, studies included must employ a longitudinal design; cross-sectional studies or analyses of only one wave of longitudinal data will be excluded. Prospective and retrospective observational cohort studies will be eligible but randomized controlled trials will not; the focus of the review is on predictors of change independent of formal prevention or intervention programming. Predictor variables will include indicators from any domain, including biological, psychological, developmental, and environmental variables, but will be required to be distinct from the outcomes (see below). Furthermore, given the focus on aging out of heavy episodic drinking, the included studies will be restricted to investigations reporting a significant reduction in drinking over time on at least one indicator (as opposed to studies reporting alcohol progression of individuals in the early portion of the emerging adult window). No demographic restrictions in terms of socioeconomic status, race, sex, gender, and orientation will be used.

### Outcome measures

The two primary outcome variables will be (1) quantity/frequency of alcohol consumed (e.g. drinks per week, drinks per month, percent drinking days) and (2) severity of alcohol-related problems. Severity of alcohol-related problems will be assessed using semi-structured clinical interviews for a diagnosis of alcohol use disorder (AUD) or questionnaires measuring functional impairment due to alcohol use (e.g. *Young Adult Alcohol Consequences Questionnaire*). In addition, due to inherent risks and health-related problems associated with heavy episodic drinking, frequency of binge episodes will also be considered as a measure of alcohol use severity. Both continuously and dichotomously operationalized outcomes will be included. To focus specifically on changes in heavy episodic drinking, we will not include studies with composite measures of polysubstance use (e.g. a latent variable comprising alcohol, tobacco, cannabis, stimulants). By doing so, we aim to be conservative in identifying predictors of developmental changes in HED specifically. To keep a clear distinction between predictors and outcomes, variables will only be able to be classified in one category or the other throughout the review. In other words, drinking quantity variables, for example, will not be included as predictors of alcohol severity outcomes. If ambiguities arise regarding whether an indicator is a predictor or an outcome, three reviewers will blindly categorize the variable and it will be designated according to the majority. No a priori secondary outcomes are proposed.

### Selection of studies

All articles retrieved during the initial search will be uploaded to Covidence, an online management system for systematic reviews for screening, or equivalent software. Using predetermined criteria, two independent reviewers will perform initial title and abstract screening to identify relevant articles. We will perform a calibration exercise to train reviewers in the use of Covidence and maximize consistency prior to screening. If additional information is needed to determine eligibility, authors of the studies will be contacted for clarification. Studies deemed ineligible will be excluded from the review, and reasons for exclusion will be noted in a flow diagram following the preferred reporting items for systematic reviews and meta-analyses (PRISMA) statement [18]. Any inter-rater disagreements on study relevance that cannot be resolved in discussion will be settled by a third reviewer.

### Data extraction and management

Data extraction will be conducted using Microsoft Excel or equivalent software. The two reviewers will independently extract data from the studies included and record details in separate Excel spreadsheets. Prior to the extraction stage, the two reviewers will perform a calibration exercise. The following information will be extracted from each study: publication details (title, author, year, journal, and country), study design (length of study, inclusion criteria, cohort age range, and number of collection waves), demographics (sample size, ethnicity, sex, self-reported racial composition [e.g. proportions of White, Asian, or Black/African American status and composition will be reported in the categories used in the original study), predictor variables (e.g. family history of alcohol use, personality traits, and expectancies), outcome measures (i.e. consumption and severity), confounding variables, narrative summary of main findings, and unadjusted and adjusted statistical results using the adjusted estimates that are identified as the primary adjusted model by the authors (*p* values, effect sizes, and confidence intervals). In the case of multiple outcomes reported within the same study, all results will be recorded to allow for later grouping of studies by outcome measurement. Any unreported or missing data will be noted, and authors will be contacted if calculating these figures from existing data is not possible. Discrepancies will be resolved through discussion or consultation with a third reviewer.

### Risk of bias of included studies

Risk of bias (RoB) of included studies will be evaluated with RoB instruments developed by the Clinical Advances through Research and Information Translation (CLARITY) tools [19]. Each domain will be judged on a 4-point Likert scale from definitely yes (low RoB) to definitely no (high RoB). Two independent reviewers will conduct RoB assessments including domains related to sampling, confidence in assessments of exposures and outcomes, and loss to follow-up. Discrepancies in judgments will be discussed, and if a consensus is not met, a third reviewer will resolve the remaining discrepancies.

### Data synthesis

Findings from the included studies will be reported in tables that summarize the fields from the extraction form and are concurrently described in narrative format. We will also meta-analyse data for all outcomes for which we have ten or more effect sizes. To determine pooled associations between risk factors and maturing out, we will combine the results using Comprehensive Meta-Analysis V2.2 (Biostat; USA), Stata V15.0 (StataCorp; USA), or other appropriate software packages. Since most studies will be observational in nature, the effect size of primary interest will be correlation or regression coefficients (e.g. Pearson’s *r*, Spearman’s *ρ*, β coefficients), but any test statistics that can be converted into an effect size will be acceptable. Effect sizes and associated standard errors will be combined using the random effects (DerSimonian and Laird) method. This is based on the assumption that there is no single ‘true’ effect size based on heterogeneity of design and measurement, but a distribution of effect sizes. Analyses will be conducted separately for unadjusted and adjusted effect estimates. Where the outcome is operationalized continuously using different data collection tools, results will be converted to standardized effect estimates. Standardized estimates that are 0.2, 0.5, and 0.8 in magnitude will be interpreted as small, medium, and large effects, respectively [20]. Where the outcome is presented as an odds ratio (OR), the log odds will be calculated prior to pooling. Heterogeneity will be assessed by visually inspecting the forest plots and *I*^2^ statistic indicating the heterogeneity of effect sizes [21]. *I*^2^ values are typically categorized as ‘modest’ (0–40%), ‘moderate’ (30–60%), ‘substantial’ (50–90%), or ‘high’ (75–100%) [21]. Due to the overlap in classification of categories, a moderate *I*^2^ in our study will be operationalized as 55% due to expected heterogeneity in non-randomized studies given methodological diversity and potential bias [21]. Further analyses to characterize the nature of the heterogeneity via subgroup analyses will be on an exploratory (and indicated as such in the completed review) due to the unknown scope and nature of possible subgroups. If quantitative pooling of results is not possible due to insufficient studies (i.e. less than 10), results will be presented narratively.

We will examine several quantitative indicators of bias. The presence of potential small study bias will be indicated by significant values on the Egger’s test [22]. Using a trim-and-fill method, imputed missing studies will be used to generate corrected estimates of the meta-analytic effect size [23]. When there are at least 10 studies identified for a pooled effect estimate, the change in published effect sizes over time will be evaluated by performing a meta-regression between publication year and effect size. Lastly, a subgroup analysis will be performed to assess the impact of RoB on each effect size when there are at least three studies identified as low and three identified as high risk of bias.

### Quality assessment

The Grading or Recommendations Assessment, Development, and Evaluation (GRADE) approach will be used to rate the quality of the aggregated evidence [24]. When meta-analysis is possible, the pooled standardized estimates or pooled RR/ORs, along with the GRADE assessment, will be presented in a Summary of Findings table.

### Evidence map

We will additionally create an ‘evidence map’ to organize the finding using a cross-tabular format and crossing specific mechanisms with different drinking outcomes. Cross-tabular formats are the most common way to present evidence maps [25], and this map will detail the number and quality of the studies on a given predictor, the combined sample size, and the outcomes measured.

## Discussion

The proposed systematic review will be reported in accordance with the PRISMA statement [18]. Any amendments made to this protocol when conducting the study will be outlined in PROSPERO and outlined in the final manuscript. The results of this systematic review are expected to provide insights into the developmental course of HED by identifying factors contributing to both successful maturing out and persistent heavy episodic drinking. Determining predictors of chronic heavy drinking patterns into adulthood will have important implications for informing early interventions. To the best of our knowledge, this systematic review will be the first conducted on both proximal and developmental contributors to heavy episodic drinking within emerging adults. Given the major health burdens associated with heavy drinking in young adults, this is an important step towards reducing alcohol-related harm. By identifying adolescents and emerging adults at-risk for prolonged HED, specific treatments could reduce their likelihood of alcohol-related problems, acute morbidity/mortality, and lifelong negative outcomes. Similarly, describing protective factors for successful maturing out will be crucial for clarifying the etiological picture of the normal trajectory of alcohol use in young people.

## Additional files

Additional file 1: PetkerT PRISMA-P Checklist: completed PRISMA-P checklist applied to this protocol (PDF 103 kb)

Additional file 2: Example search strategy: example of the search strategy to be used in the review (PDF 61 kb)

## Data Availability

Not applicable.
