# Supplementary material for: Predictors of aging out of heavy episodic drinking in emerging adults: a systematic review protocol
Source: Syst Rev. 2019 Sep 4;8:230. doi: 10.1186/s13643-019-1139-9 (PMC6724318; doi:10.1186/s13643-019-1139-9)
Supplement: Supplementary file 2 — Example search strategy: example of the search strategy to be used in the review (PDF 61 kb) [file 13643_2019_1139_MOESM2_ESM.pdf]

**Additional File 2: Example search strategy**

Draft Public Library of Medicine (PubMed) free text search:

1. Alcohol
2. Matur* or Age) adj2 Out
3. Transition out
4. Transitional
5. Age Factors
6. Emerging adult
7. College student and university student
8. Or/2-7
9. Longitudinal
10. Growth
11. Trajector*
12. Mixture model
13. Prospective stud*
14. Theoretical model
15. Retrospective
16. Or/9-15
17. Heavy episodic drinking
18. Alcoholism
19. Binge drinking
20. Alcohol us*
21. Alcohol misuse
22. Alcohol abus*
23. Drug us*
24. Or/17-23
25. **Search: 1 AND 8 AND 16 AND 24**

The preceding example reflects: (1) Drug + (2) Developmental Period + (3) Methodology /Design + (4) Problematic Level

No planned limits will be imposed on the search.
